# Suppression of lncRNA NLRP3 inhibits NLRP3-triggered inflammatory responses in early acute lung injury

**DOI:** 10.1038/s41419-021-04180-y

**Published:** 2021-10-01

**Authors:** Deqiang Luo, Wei Dai, Xiaojin Feng, Chengzhi Ding, Qiang Shao, Rui Xiao, Ning Zhao, Wei Peng, Ying Yang, Yamei Cui, Fen Liu, Kejian Qian

**Affiliations:** 1 Department of Intensive Care Unit, The First Affiliated Hospital of Nanchang University, No. 17 Yongwaizheng Street, Dong Lake District, Nanchang, Jiangxi Province 330000 China; 2 Medical Innovation Center, The First Affiliated Hospital of Nanchang University, Nanchang, China; 3 Department of Intensive Care Unit, The Fifth People’s Hospital of Shangrao City, No. 1 Jiannan Road, Xin Zhou District, Shangrao, Jiangxi Province 334000 China; 4 Department of Anesthesiology, The Second Affiliated Hospital of Nanchang University, No. 1 Minde Road, Dong Lake District, Nanchang, Jiangxi Province 330000 China

**Keywords:** Bacterial infection, Inflammasome

## Abstract

Acute lung injury (ALI) is a common lung pathology that is accompanied by alveolar macrophage (AM) activation and inflammatory response. This study investigated the role of the long non-coding RNA NONRATT004344 (hereafter named lncRNA NLRP3) in regulating the Nod-like receptor protein 3 (NLRP3)-triggered inflammatory response in early ALI and the underlying mechanism as well. We established LPS-induced ALI models to explore their interactive mechanisms in vitro and in vivo. Luciferase reporter assays were performed to determine that miR-138-5p could bind to lncRNA NLRP3 and NLRP3. We observed increased lncRNA NLRP3 expression, decreased miR-138-5p expression, NLRP3 inflammasome activation, and upregulated caspase-1, IL-1β, and IL-18 expression in the LPS-induced ALI model. Furthermore, lncRNA NLRP3 overexpression activated the NLRP3 inflammasome and promoted IL-1β and IL-18 secretion; the miR-138-5p mimic abolished these effects in vivo and in vitro. Consistently, miR-138-5p inhibition reversed the effects of lncRNA NLRP3 silencing on the expression of NLRP3-related molecules and inhibition of the NLRP3/caspase-1/IL-1β signalling pathway. Mechanistically, lncRNA NLRP3 sponging miR-138-5p facilitated NLRP3 activation through a competitive endogenous RNA (ceRNA) mechanism. In summary, our results suggested that lncRNA NLRP3 binding miR-138-5p promotes NLRP3-triggered inflammatory response via lncRNA NLRP3**/**miR-138-5p/NLRP3 ceRNA network (ceRNET) and provides insights into the treatment of early ALI.

## Introduction

Acute lung injury (ALI)/mild acute respiratory distress syndrome (ARDS) in Berlin definition, which is accompanied by alveolar macrophage (AM) activation and inflammatory response, often progresses to an inflammatory storm that develops into severe ARDS and patient death [1, 2]. The innate immune system and macrophages perform a critical defensive function to protect the host against external stimuli such as lipopolysaccharide (LPS) in the early stage of ALI [3]. The imbalance of innate immune regulatory mechanisms results in systemic inflammatory response syndrome, multiorgan failure, or even death [4, 5]. Therefore, the molecular mechanisms underlying the regulation of host innate immunity need to be identified and are of great significance for identifying new molecular biomarkers and finding effective ALI therapies.

Nod-like receptor protein 3 (NLRP3) is an essential functional component of the NLRP3 inflammasome complex and it contains the nucleotide-binding domain and repeated leucine-rich sequences [6]. NLRP3 inflammasome activation effectively initiates the innate immune response by promoting the maturation and secretion of inflammatory molecules, such as interleukin (IL)-1β and IL-18 in the cytoplasm [7, 8]. NLRP3 inflammasome activation is involved in the early pathophysiological process of ALI and influences early inflammatory responses by assembling inflammatory components into multiprotein complexes in the cytoplasm [9]. Up to now, the researchers have found a two-signal model for NLRP3 activation. One signal is the priming step that is triggered by endogenous molecules or microbial components. The second activation signal is induced by most or all NLRP3 molecules, which cleave pro-IL-1β and pro-IL-18 to generate active forms that are released as inflammatory cytokines [10]. In the NLRP3/IL-1β pathway, IL-1β and IL-18 are generally considered critical triggers of lung dysfunction during sepsis [11, 12]. However, the exact molecular mechanisms by which NLRP3 activation promotes IL-1β/IL-18 secretion during LPS-induced ALI remain unknown.

Non-coding RNAs (long non-coding RNA (lncRNA), microRNA (miRNA), and circular RNA (circRNA), *etc*.) are a group of endogenous cellular RNAs without protein-coding capacity; in recent years, these molecules have been shown to play essential roles in the pathophysiological processes of multiple diseases [13]. Numerous studies have found that lncRNAs regulate various physiological and pathological processes, such as inflammatory diseases, cardiovascular diseases, inflammatory responses, neuroinflammation, and Alzheimer’s disease, by targeting the NLRP3 inflammasome [14–24]. miR-138-5p has been found to be a tumour suppressor in bladder [25], non-small cell lung [26], colorectal [27], malignant glioblastoma [28], and pancreatic cancer [29]. Our previous study found that miR-138-5p plays a vital role in cognitive impairment by targeting NLRP3 [30]. We also predicted that with RNA sequencing (RNA-seq) and bioinformatics analysis, the lncRNA NONRATT004344/miR-138-5p/NLRP3 ceRNA network (ceRNET) could regulate NLRP3-triggered inflammatory responses in ALI [31]. However, further experimental verification is required.

Our study found that lncRNA NONRATT004344 (hereafter named lncRNA NLRP3) and NLRP3 expression was increased in LPS-induced ALI, and RNA-seq and bioinformatics analysis revealed there was a target-regulatory relationship between these molecules. To validate the role of lncRNA NLRP3 in regulating the NLRP3-triggered inflammatory response in ALI and to explore the underlying mechanism, we established the ALI models in vitro and in vivo. We confirmed that lncRNA NLRP3 and NLRP3-related molecule expression was upregulated in LPS-induced ALI, whereas miR-138-5 expression was downregulated. Our study found that lncRNA NLRP3 sponges miR-138-5p to facilitate the NLRP3-triggered inflammatory response via the lncRNA NLRP3**/**miR-138-5p/NLRP3 ceRNET, and lncRNA NLRP3 and miR-138-5p provide new targets for the treatment of ALI.

## Materials and methods

### LPS-induced AM cell modelling

The *Rattus norvegicus* (NR) 8383 AM cell line was purchased from the Chinese Academy of Sciences Cell Bank (Shanghai city, China). Short tandem repeat profiling (CASCB, Shanghai city, China) was used to authenticate the NR8383 AM cell line. Before use, NR8383 AM cells were needed to differentiate into macrophages by treatment with 25 ng/ml phorbol 12-myristate 13-acetate (Sigma) overnight and cultured in Ham’s F-12K medium containing 15% (v/v) fetal bovine serum (FBS). Inflammatory responses were induced by treating the NR8383 AM cells with 1 μg/μl LPS (*Escherichia coli* 055: B5, Sigma-Aldrich). The negative control group (NC) was treated with the same volume of phosphate-buffered saline (PBS) (Solarbio Life Science, P1010, Beijing city, China) alone. The LPS group was treated with 1 μg/μl LPS alone for 2 and 9 h. Three groups of AM samples were then sent for RNA-seq. We randomly assigned the cells into 12 groups in subsequent experiment: the NC, LPS, Lv-lncRNA NLRP3 + LPS, Lv-lncRNA NLRP3 NC + LPS, silncRNA NLRP3 + LPS, silncRNA NLRP3 NC + LPS, miR-138-5p inhibitor + LPS, miR-138-5p inhibitor NC + LPS, miR-138-5p mimic + LPS, miR-138-5p mimic NC + LPS, silncRNA NLRP3 + miR-138-5p inhibitor + LPS, and Lv-lncRNA NLRP3 + miR-138-5p mimic + LPS. AM cells were seeded in six-well plates with 2 × 10^5^ cells/well prior to the transfection. According to the instructions (RiboBio Co., Ltd, Guangzhou, China), the working concentration of silncRNA NLRP3, silncRNA NLRP3 + NC, miR-138-5p inhibitor, miR-138-5p mimic, miR-138-5p inhibitor NC, or miR-138-5p mimic NC was kept at 100 nM and then they were mixed with its riboFECTtm CP Reagent, respectively. The AM cells were transfected for 72 h and subsequently treated by LPS (1 μg/mL) for 6 h, and then they were collected for RNA or protein isolation. Inflammatory factors of the collected supernatant medium were measured with enzyme-linked immunosorbent assay (ELISA). The AM cells were collected for western blot analysis, ELISA, and quantitative reverse-transcriptase PCR (qRT-PCR) (*n* = 6 per group).

### Animals

Forty-eight 4-week-old male SD rats weighing 90–110 g were purchased from Jiangxi University of Traditional Chinese. We randomly divided the rats into eight groups (the NC, LPS, si-r-lncRNA NLRP3, Lv-lncRNA NLRP3 + LPS, miR-138-5p agomiR + LPS, antagomiR-138-5p + LPS, Lv-lncRNA NLRP3 + agomiR-138-5p + LPS, and si-r-lnc NLRP3 + antagomiR-138-5p + LPS; *n* = 6 rats in each group). Before the experiment, the rats were not drinking water for 4 h and fasting for 12 h. The Ethical Committee of Nanchang University approved all the animal procedures that followed the Experimental Animal Principles and Guidelines of Nanchang University. We performed the invasive procedures with pentobarbital anaesthesia to minimize suffering and killed all the animals by inhaling excessive amounts of isoflurane anaesthetic.

### The LPS-induced ALI rat modelling

The rats were anaesthetized by inhaling isoflurane and intraperitoneal injection of 3% sodium pentobarbital (150 mg/kg). The rats were pretreated for 72 h with PBS, si-r-lncRNA NLRP3 (2.5 nmol/100 μl), Lv-lncRNA NLRP3 (1 × 10^7^ TU/100 μl), agomiR-138-5p (2.5 nmol/100 μl), antagomiR-138-5p (2.5 nmol/100 μl), Lv-lncRNA NLRP3 (1 × 10^7^ TU/50 μl) + agomiR-138-5p (2.5 nmol/50 μl), or si-r-lnc NLRP3 (2.5 nmol/50 μl) + antagomiR-138-5p (2.5 nmol/50 μl). The ALI models of the rats were established by intratracheal instillation with 5 mg/kg/100 μl LPS. The NC group received intratracheal instillation of PBS alone. The LPS group was intraperitoneally injected with LPS alone (5 mg/kg/100 μl) and killed 6 h later. The rats in the other groups were killed 6 h after LPS administration.

### RNA transfection and primers

The AM cells were pretreated for 48 h before RNA extraction and 72 h before protein extraction (*n* = 6). The cells were treated with 50 nmol/l silncRNA NLRP3, 50 nmol/l silncRNA NLRP3 NC, 1 × 10^7^ TU/100 μl Lv-lncRNA NLRP3, 1 × 10^7^ TU/100 μl Lv-lncRNA NLRP3 NC, 50 nmol/l miR-138-5p mimics, 50 nmol/l miR-138-5p mimics NC, 50 nmol/l miR-138-5p inhibitor, 50 nmol/l miR-138-5p inhibitor NC, 1 × 10^7^ TU/100 μl Lv-lncRNA NLRP3 + 50 nmol/l miR-138-5p mimics, and 100 nmol/l silncRNA NLRP3 (100 nmol/l) + 50 nmol/l miR-138-5p inhibitor. Then, the AM cells in the LPS group were treated with LPS (1 μg/μl) for 6 h. RiboBio Co. Ltd (Guangzhou City, China) synthesized silncRNA NLRP3, miR-138-5p mimics, and inhibitor. Lv-LncRNA was obtained from GENE Co. Ltd (Shanghai City, China). The sequences of the silncRNA targeting NLRP3 are as follows:

silncRNA NLRP3_001: 5′-CAGAAGCTTGGAGGATAGA-3′;

silncRNA NLRP3_002: 5′-CACTCTCCTTTTCTCAAAG-3′;

silncRNA NLRP3_003: 5′-GATAGAGGCTCTTTTCTTT-3′.

After pre-experiments, we finally selected silncRNA NLRP3_003 as the most efficient transfection sequence. The target sequence of silncRNA NLRP3 in rat lung was listed as follows:

si-r-lncRNA NLRP3_003: 5′-GATAGAGGCTCTTTTCTTT-3′.

The sequences for PCR primers and siRNAs were shown in Supplementary Table 1.

### Quantitative RT-PCR

Total RNA extraction from AM cells was used for qRT-PCR with the TRIzol reagent (Invitrogen, Carlsbad, CA, USA). qRT-PCR analysis was performed with an SYBR Green Kit (Takara, Shanghai City, China) according to the instructions. The qRT-PCR mixtures contained 10.0 μL of 2 × qPCR mix, 2.0 μL of Rox Reference Dye, 4.4 μL of ddH_2_O, 1.6 μl of primers (7.5 μM), and 2.0 μL of the reverse-transcription product. Optimization of the qRT-PCR amplification conditions was as follows: 94 °C for 1.5 min, 30 cycles of each at 94 °C for 20 s, 60 °C for 20 s, and 72 °C for 60 s. β-Actin was used as the internal control.

### Intracellular fractionation

First, the intracellular location of lncRNA NLRP3 was analysed by Locate-R based on a previous study [32]. Then, the total RNA profile of cytoplasmic and nuclear was isolated using NE-PER™ Nuclear Cytoplasmic Extraction Reagents (78833, Thermo Fisher Scientific, Shanghai City, China) and determine the subcellular localization of lncRNA NLRP3 using an RNeasy Midi Kit (Qiagen, Hilden, Germany). The extracted RNA expression levels of lncRNA NLRP3 and β-actin (nuclear control transcript and cytoplasmic control transcript) from each fraction were analysed by qRT-PCR.

### RNA immunoprecipitation

An EZ-Magna RNA immunoprecipitation (RIP) kit (Millipore, MA) was used to perform the RIP assay. Briefly, NR8383 AM cells (1 × 10^7^) were collected and incubated with RIP lysis buffer. The precleared lysates were used for RIP with anti-NLRP3 (Boster Biotech; BA3677, 1 : 200, China) and rabbit isotype control IgG antibodies. RNA was isolated and purified using an acid phenol/chloroform method.

### Lentivirus gene delivery

Lentivirus infection was carried out as previously described [31]. Lentiviruses overexpressing lncRNA NLRP3 were constructed by GeneChem Group (Gene Co. Ltd, Shanghai City, China). AM cells were cultured in a medium with Ham’s F-12K containing 15% (v/v) FBS. The RNA extraction groups were transfected with the indicated lentiviruses for 48 h and the protein extraction groups were transfected with the indicated lentiviruses for 72 h in the culture medium. Then, the medium was replaced by fresh Ham’s F-12K containing 15% FBS for 24 h.

### Western blotting

Total protein extraction from NR8383 AM cells (5 × 10^6^ cells) and rat lungs were performed with radioimmunoprecipitation assay lysis and extraction buffer (Thermo Fisher Scientific, Shanghai City, China). We used 10% SDS-polyacrylamide gel electrophoresis to separate the protein samples and transferred them to polyvinylidene difluoride membranes (Millipore, Billerica, MA, USA). Next, the membranes were blocked with 5% non-fat milk for 1 h at room temperature (RT) and incubated with primary antibodies for 12 h at 4 °C. Then, the membranes were incubated with secondary antibodies at RT for 4 h. We used glyceraldehyde 3-phosphate dehydrogenase (GAPDH) as the internal reference. All blots were visualized by the enhanced chemiluminescence kit assay (Millipore, Billerica, MA, USA) and were developed with a Bio-Rad Gel Doc EZ imager (Bio-Rad, USA), and the band intensities were analysed by ImageJ software (NIH Image analysis). The primary antibodies used in this experiment included rabbit polychonal anti-NLRP3 (1 : 500; Cell NBP2-12446, Novus, USA), anti-GAPDH (1 : 2000; Cell Signaling Technology, USA), and anti-caspase-1 (1 : 2000, ab1872, Abcam, USA).

### Enzyme-linked immunosorbent assay

According to the product manual, an ELISA assay was performed to detect the levels of the inflammatory cytokines IL-18 and IL-1β. In brief, AM cells were seeded in 24-well plates at a density of 2 × 10^5^ cells/mL. Culture Petri dishes supernatants from each group were collected at the indicated time points. The levels of the inflammatory cytokines (IL-18 and IL-1β) in the supernatants (100 μl) were analysed using R&D Systems ELISA kits (Minneapolis, MN, USA). The absorbance (A450) was measured at 450 nm to calculate the concentration of cytokines by regression analysis of a standard curve.

### Detection of myeloperoxidase activity

To assess lung neutrophil infiltration, the myeloperoxidase (MPO) activity of lung tissue homogenates mixed with thiobarbituric acid in the supernatant was detected and was expressed as activity per gram of lung tissue [33].

### Luciferase assay

The mutant-type and wild-type miR-138-5p-binding sites in the 3′-untranslated regions (3′-UTRs) of lncRNA NLRP3 and NLRP3 were cloned into the pmirGLO vector (Promega, CA, USA) following the manufacturer’s protocol. Cells overexpressing with NLRP3-Wt or NLRP3-Mut, and lncRNA-NLRP3-Wt or lncRNA-NLRP3-Mut were transfected with miR-138-5p NC or miR-138-5p mimics. At 48 h after transfection, the dual-luciferase reporter assay system (E1910, Promega, CA, USA) was used to measure Firefly and *Renilla* luciferase activities following the manufacturer’s instructions.

### Bioinformatics analyses

We analysed the evolutionary conservation of lncRNA NLRP3 via the University of California Santa Cruz (UCSC) Genome Browser (genome.ucsc.edu). The protein-coding capacity of lncRNA NLRP3 was analysed using the Institute of Bioinformatics and System Biology, the National Chiao Tung University of Taiwan, for Bioinformatics (http://regrna2.mbc.nctu.edu.tw/index.html) [34]. We searched the particularly conserved regions within the 2 kb upstream of the promoter regions of lncRNA NLRP3 and found the promoter sequences of lncRNA NLRP3 in the Ensembl database (http://asia.ensembl.org/index.html) [35, 36]. Targetscan (http://www.targetscan.org/mmu_72) [37], the miRbase (http://www.mirbase.org) [38], Starbase (http://starbase.sysu.edu.cn/) [39], RNA-inter (http://www.rna-society.org/rnainter/IntaRNA.html) [40], and RNAhybrid (https://bibiserv.cebitec.uni-Bielefeld.de/rnahybrid/) results revealed that there were miR-138-5p sites in the lncRNA NLRP3 sequence region. Moreover, the binding sites of miR-138-5p in the NLRP3 gene promoter were identified using the same method mentioned above.

### Lung wet/dry ratio

The lung oedema was identified by calculating the lung wet/dry (W/D) ratio. Immediately after killing the rats, the wet right lungs of rats from each group were weighed and then dried in an incubator at 60 °C for 24 h. Dry weight was measured and W/D ratios were calculated.

### Histopathological analysis

Haematoxylin and eosin (HE) staining was used to analyse the histopathological analysis of lung tissues. The lung tissue of rats from each group was immediately fixed in 4% paraformaldehyde, processed, and embedded in paraffin. Then 5–7 μm-thick sections were acquired and subjected to HE staining, and HE-stained images were captured using an Olympus IX71 microscope (Olympus, Tokyo, Japan). Lung injury scores were calculated [41]. The sections were analysed to assess inflammatory cell infiltration, epithelial desquamation, oedema, and haemorrhage. The degree of each characteristic was scored as 3 (prominent), 2 (moderate), 1 (mild), and 0 (absence).

### Albumin concentration measurement

Bronchoalveolar lavage fluid (BALF) from each group was collected [1 mL of PBS (1 mmol/L KH_2_PO_4_ pH 7.4, 3 mmol/L KCl, 140 mmol/L NaCl, 6 mmol/L Na_2_HPO_4_, and PBS)] (*n* = 6 rats per group). Collectively, 90% (27 mL) of the total administered volume was recovered from each rat. The collected BALF was used to measure albumin levels (EK0592 and Solarbio Life Science; SEKR-0009; ELISA; Boster Biotech).

### Hoechst 33342 staining

AM cells were treated as indicated. Sufficient Hoechst 33342 (Solarbio Life Science; AR-0039) staining solution was added to stain cells by incubating for 30 min in the dark. After being washed twice with PBS, the cells were observed and imaged with a fluorescence microscope (Olympus, Tokyo, Japan).

### Immunohistochemical staining

The NLRP3 activation levels in the lung tissues were measured by immunohistochemical (IHC) staining, which was performed following a previously described method [42]. First, the lung tissue sections were subjected to induced antigen retrieval in citrate buffer at 100 °C (0.01 M, pH 6.0) and then immersed in 3% hydrogen peroxide solution for 10 min at RT to quench endogenous peroxidase activity. After blocking with 5% bull serum albumin, the tissues were incubated with anti-NLRP3 antibody (Boster Biotech; BA3677, 1 : 200, China) overnight at 4 °C. Afterward, the sections were rinsed with PBS three times and incubated with anti-rabbit IgG secondary antibodies (1 : 200, Boster, Wuhan, China) at RT for 1 h, and then were viewed by 3,3′-diaminobenzidine solution and counterstained with hematoxylin. The sections were visualized and analyzed by a light microscope (Olympus, Tokyo, Japan) equipped with an imaging system and ImageJ software.

### Immunofluorescence staining

Rat lung tissues containing the hippocampus were heated in citrate buffer (pH 6.0) at 100 °C for antigen retrieval and then placed on glass chamber slides fixed in 4% paraformaldehyde, blocked in PBS containing 2% bovine serum albumin with 0.2% Triton X-100 for 30 min at RT. Then, the rat lung tissues were coincubated with antibodies against CD68 (BA3638, Boster Co. Ltd, China) and NLRP3 antibody (1 : 50, BA3677, Boster, Wuhan, China) overnight at 4 °C. An Alexa Fluor 594-conjugated phalloidin was added and incubated with an appropriate secondary antibody at 25 °C in the dark. 4′,6-Diamidino-2-phenylindole was used to stain the nuclei. IF images were acquired with an Olympus IX71 microscope (Olympus, Tokyo, Japan).

### Statistical analysis

The acquired data were analysed using the GraphPad Prism software (version 9.0) and are presented as the means ± SE of six measurements. Student’s *t*-test and one-way analysis of variance were used to assess the differences among groups. All statistical tests were two-tailed and *P* < 0.05 was considered to indicate statistically significant differences.

## Results

### The expression of lncRNA NLRP3 and its target NLRP3 is upregulated in ALI

**Fig. 1:**
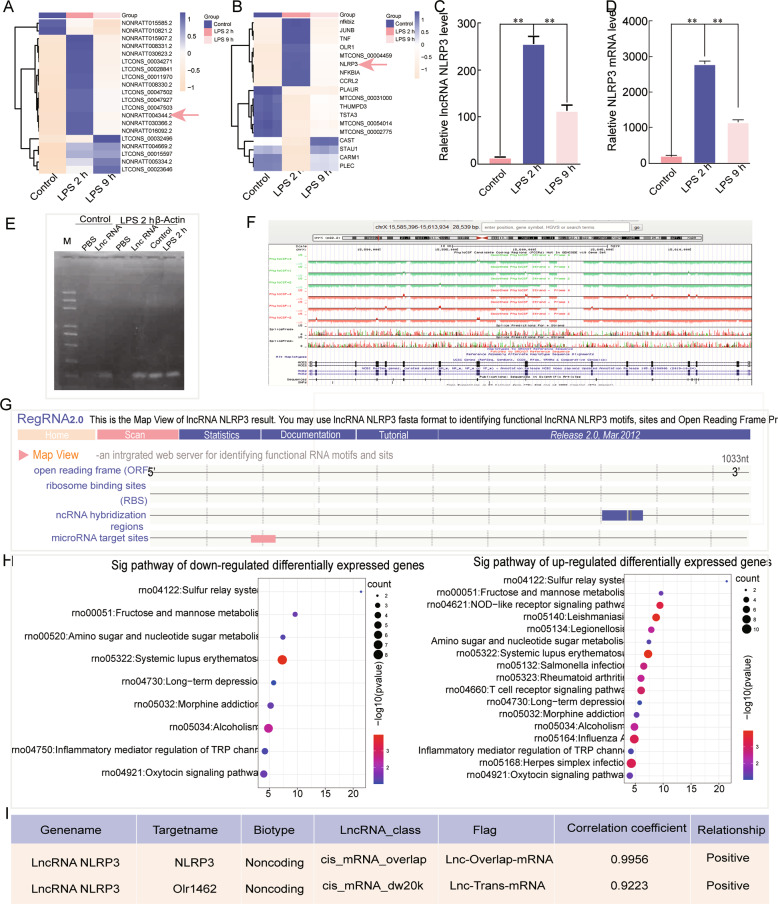
Dysregulated transcription of lncRNA NLRP3 and NLRP3 in LPS-treated NR8383 AM cells as determined by RNA-seq and bioinformatics analysis. **A** The heat map lists the top 20 differentially expressed lncRNAs and mRNAs in NR8383 AM after treatment with PBS, LPS for 2 h, and LPS for 9 h. **A**, **B** RNA-seq analysis shows the quantified gene expression of lncRNA NLRP3 and NLRP3 in AM cells in the negative control, LPS 2 h, and LPS 9 h groups. **C**, **D** Agarose gel electrophoresis analysis shows the quantified expression of lncRNA NLRP3 and NLRP3 in NR8383 cells. β-Actin served as the control. **E** The conservation of lncRNA NLRP3 was predicted and analysed by the UCSC Genome Browser. **F** The lncRNA NLRP3 potential protein-coding and binding sites were analysed with RNA 2.0 tools. **G** The results show that lncRNA NLRP3 has no protein-coding capability. **H** Gene Ontology and Kyoto Encyclopedia of Genes and Genomes pathway analysis were used to analyze differentially expressed genes. **I** The relationship between lncRNA NLRP3 and NLRP3, and the correlation coefficient is listed. *P < 0.05; **P < 0.01; ***P < 0.001; NS, no statistically significant difference.

RNA-seq was used to analyse the whole transcriptome of LPS-treated NR8383 AM cells. The histogram revealed the top 20 differentially expressed lncRNAs and mRNAs in LPS-treated AM cells compared with control cells (Fig. 1A, B and Supplementary Excels 2–5). The expression of lncRNA NLRP3 and NLRP3 was highest in the AM cells treated with LPS for 2 h compared to the NC and cells treated with LPS for 9 h (*P* < 0.05, Fig. 1C, D). The agarose gel electrophoresis results validated the above RNA-seq results (Fig. 1E). The conservation of lncRNA NLRP3 was analysed with the UCSC Genome Browser and the results showed it to be an exceedingly conserved species (Fig. 1F). The bioinformatics results showed that lncRNA NLRP3 cannot encode protein and has a miRNA-binding site (Fig. 1G). Gene Ontology and Kyoto Encyclopedia of Genes and Genomes pathway analyses of the differentially expressed pathways in ALI showed pathways associated with inflammation: the NOD-like receptor signalling pathway and the regulation of Transient Receptor Potential (TRP) (channels by inflammatory mediators) (Fig. 1H). We further found a sequence regulatory relationship between lncRNA NLRP3 and NLRP3 by sequence alignment and coexpression net analysis, and the weighted correlation coefficient between these molecules was 0.9956 (Fig. 1I). These results show that LPS could increase lncRNA NLRP3 and NLRP3 expression in the early stage of ALI and there is a target-regulatory relationship between these molecules.

### Effects of LPS on the expression of lncRNA NLRP3, miR-138-5p, NLRP3, caspase-1, IL-1β, and IL-18 in early ALI

**Fig. 2:**
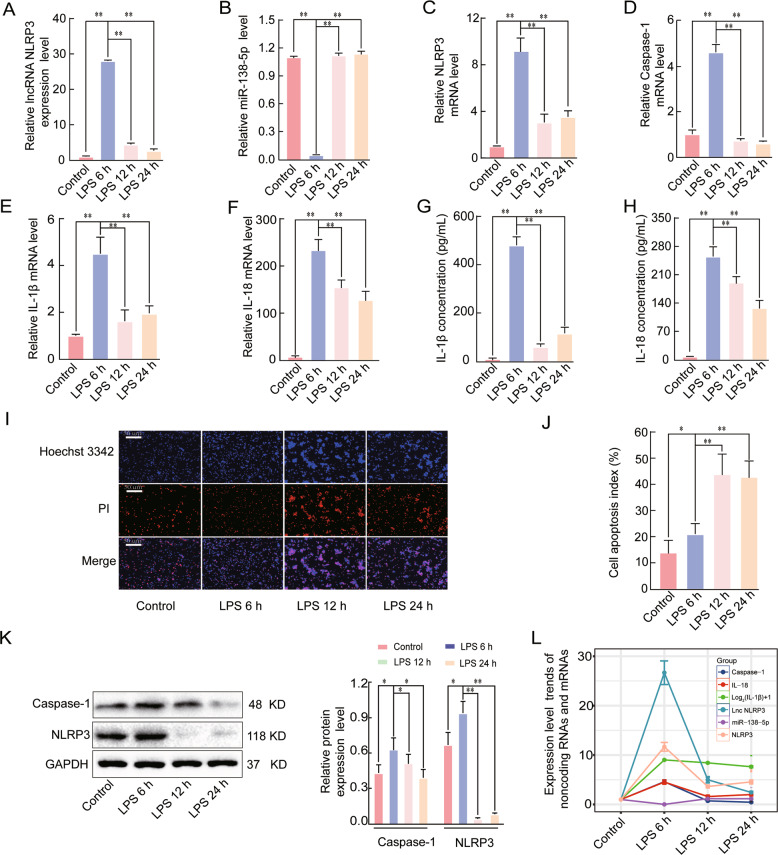
Effects of LPS on the lncRNA NLRP3, miR-138-5p, NLRP3, Caspase-1, IL-1β, and IL-18 expression levels in early ALI. A qRT-PCR assay was used to analyse the mRNA expression of **A** LncRNA NLRP3, **B** miR-138-5p, **C** NLRP3, **D** Caspase-1, **E** IL-1β, and **F** IL-18 in LPS-induced ALI. β-Actin was used as the reference gene. **G**, **H** ELISA analysis of the IL-1β and IL-18 levels in the culture supernatant. Cell apoptosis was determined by Hoechst 33342 and PI dual staining assays (**I**) and counted (**J**). The expression of NLRP3 and caspase-1 in the NR8383 AM cells from the four groups was analysed by western blotting (**K**). **L** Expression trends of lncRNA NLRP3, NLRP3, caspase-1, IL-1β, IL-18, and miR-138-5p in the negative control group and groups treated with LPS for 6, 12, and 24 h. The data are presented as mean ± SE (*n* = 6). *P < 0.05; **P < 0.01; ***P < 0.001; NS, no statistically significant difference.

We established an ALI model with the LPS-stimulated NR8383 AM cells, to investigate the timepoint at which the most significant effects on lncRNA NLRP3, miR-138-5p, caspase-1, and NLRP3 expression occur. The expression levels of lncRNA NLRP3, miR-138-5p, NLRP3, caspase-1, IL-1β, and IL-18 were measured by qRT-PCR. LPS stimulation for 6 h led to the most significant increase in the expression levels of lncRNA NLRP3, caspase-1, IL-18, IL-1β, and NLRP3 compared to stimulation with the NC group, LPS for 12 h, and LPS for 24 h, whereas miR-138-5p expression was most significantly downregulated at this timepoint (*P* < 0.05, Fig. 2A–F). We further investigated the expression levels of IL-1β and IL-18 in the supernatants by ELISA, and the results were consistent with the qRT-PCR results (*P* < 0.05, Fig. 2G, H). In addition, Hoechst 3342 staining showed that LPS significantly induced apoptosis at 6, 12, and 24 h (*P* < 0.05, Fig. 2I, J) compared with the NC. The protein expression levels of NLRP3 and caspase-1 also confirmed the qRT-PCR results (*P* < 0.05, Fig. 2K). These results showed that lncRNA NLRP3, NLRP3, caspase-1, IL-1β, and IL-18 expression were most significantly increased after 6 h of LPS treatment and miR-138-5p expression was most significantly decreased at this timepoint (Fig. 2L). These results indicated that 6 h for LPS stimulation was the optimal time to study the NLRP3-triggered inflammatory response in early ALI.

### LncRNA NLRP3 regulates the inflammatory response during ALI through NLRP3 inflammasomes

**Fig. 3:**
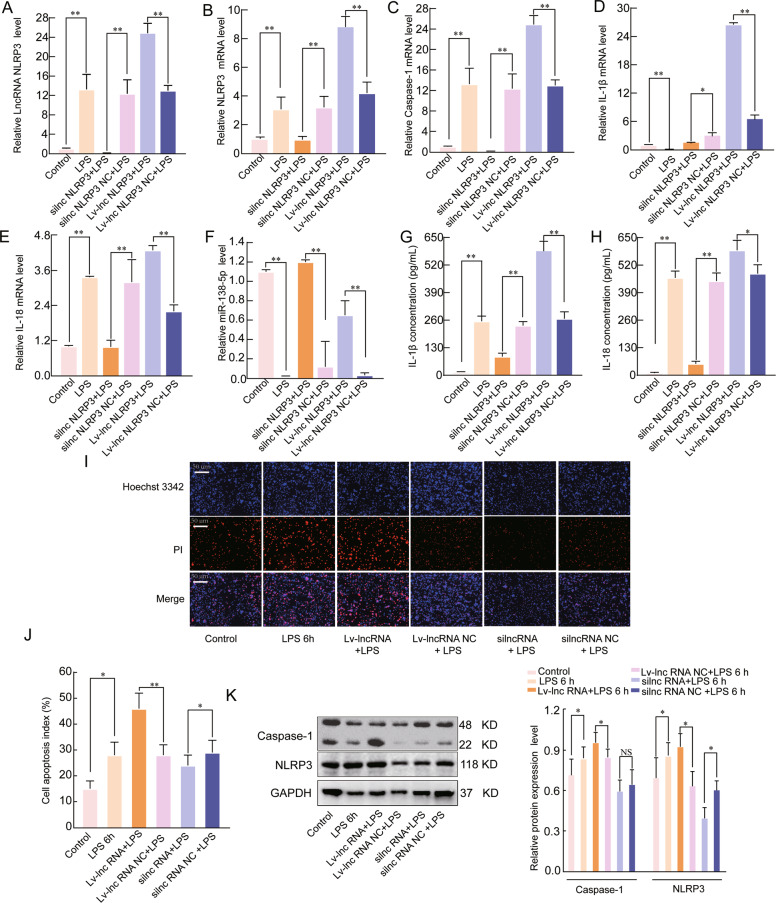
LncRNA NLRP3 regulates the inflammatory response during ALI through NLRP3 inflammasomes. A qRT-PCR assay was used to analyse the mRNA expression of **A** lncRNA NLRP3, **B** NLRP3, **C** Caspase-1, **D** IL-18, **E** IL-1β, and **F** miR-138-5p in LPS -induced ALI. β-Actin was used as the reference gene. **G**, **H** ELISA was used to analyse the IL-1β and IL-18 levels in the culture supernatants. **I**, **J** Cell apoptosis was determined by Hoechst 33342 and PI dual staining assays (**I**) and counted (**J**). **K** Western blotting was used to analyse the protein expression of NLRP3 and caspase-1 after lncRNA NLRP3 overexpression in the cytoplasm. The data are presented as mean ± SE (*n* = 6). *P < 0.05; **P < 0.01; ***P < 0.001; NS, no statistically significant difference.

To investigate whether lncRNA NLRP3 regulates the inflammatory response associated with LPS-induced ALI through NLRP3 inflammasomes, we either silenced and overexpressed lncRNA NLRP3 in NR8383 AM cells. The qRT-PCR results showed that the expression of lncRNA NLRP3, caspase-1, IL-18, IL-1β, and NLRP3 was increased under lncRNA NLRP3 overexpression conditions, whereas miR-138-5p expression was significantly decreased. However, the mRNA expression levels of lncRNA NLRP3, caspase-1, IL-18, IL-1β, and NLRP3 were suppressed, and miR-138-5p expression was significantly increased when lncRNA NLRP3 was knocked down (*P* < 0.05, Fig. 3A–F). The levels of IL-18 and IL-1β in the supernatants were consistent with the qRT-PCR results (*P* < 0.05, Fig. 3G, H). Hoechst 3342 staining showed that Lv-lncRNA NLRP3 enhanced the LPS-induced cell apoptosis, and that this effect was weakened by silncRNA NLRP3 silencing (*P* < 0.05, Fig. 3I, J). We also confirmed the above findings by examining NLRP3 and caspase-1 protein expression (*P* < 0.05, Fig. 3K). These data indicated that lncRNA NLRP3 could promote the inflammatory response associated with ALI by regulating NLRP3 inflammasome activation.

### LncRNA NLRP3 functions as a sponge for miR-138-5p

**Fig. 4:**
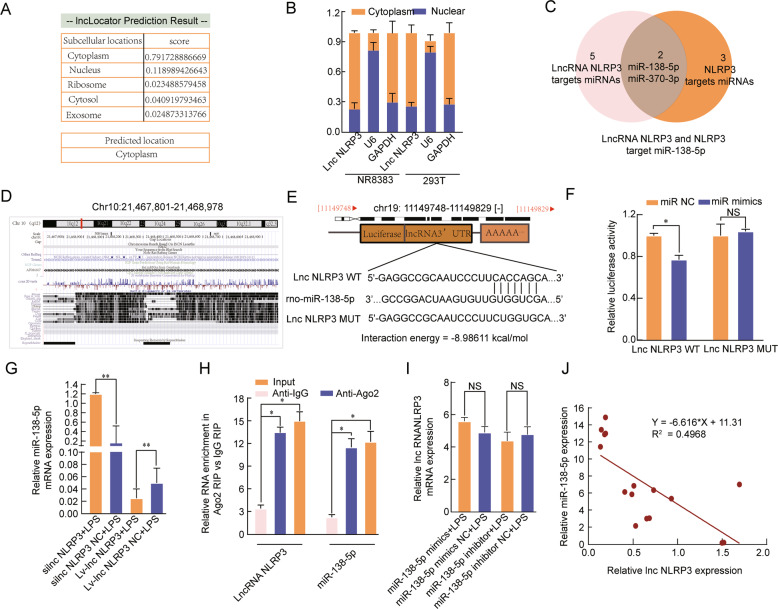
LncRNA NLRP3 functions as a sponge for miR-138-5p. **A** LncLocator was used to investigate the distribution of lncRNA NLRP3. **B** LncRNA NLRP3 was mainly located in the cytoplasm. **C** Venn diagram of miRDB predicting miR-138-5p and miR-370-3p sponged by lncRNA NLRP3 and NLRP3. **D**, **E** The predicted miR-138-5p-binding sites in the lncRNA NLRP3 3′-UTR. **F** miR-138-5p mimics notably reduced the luciferase activity of the lncRNA NLRP3-Wt group. **G** silncRNA NLRP3 significantly increased miR-138-5p expression; however, overexpression of lncRNA NLRP3 reduced miR-138-5p expression in LPS-treated NR8383 AM cells. **H** RIP assays revealed that Ago2-containing beads enriched the expression of miR-138-5p and NLRP3. **I** The miR-138-5p inhibitor and miR-138-5p mimics had no effects on lncRNA NLRP3 expression in LPS-treated NR8383 AM cells. The data are presented as mean ± SE (*n* = 6). **J** LncRNA NLRP3 expression was negatively correlated with miR-138-5p expression in LPS-treated NR8383 AM cells. *P < 0.05; **P < 0.01; ***p < 0.001; NS, no statistically significant difference.

To determine the underlying mechanism by which lncRNA NLRP3 regulates NLRP3, we first explored the distribution of lncRNA NLRP3 by LncLocator. The results showed that the vast majority of lncRNA NLRP3 was located in the cytoplasm (Fig. 4A), which was further confirmed by qRT-PCR (*P* < 0.05, Fig. 4B). The bioinformatic analysis results indicate that lncRNA NLRP3 binds to a conserved target site on miR-138-5p with a high free-binding energy and miR-138-5p is highly conserved among species (Fig. 4C–E). A dual-luciferase reporter assay demonstrated that miR-138-5p mimics markedly suppressed the luciferase activity in the lncRNA NLRP3-Wt groups (*P* < 0.05, Fig. 4F). Consistently, miR-138-5p expression was significantly increased in LPS-treated NR8383 AM cells in which lncRNA NLRP3 was knocked down; however, miR-138-5p expression was significantly reduced when lncRNA NLRP3 was overexpressed (*P* < 0.05, Fig. 4G). The RIP assay results confirmed that NLRP3 directly interacts with miR-138-5p (*P* < 0.05, Fig. 4H). There was no difference in the expression of lncRNA NLRP3 between miR-138-5p inhibitor and miR-138-5p mimic conditions, which suggests that miR-138-5p exerts no regulatory effect on lncRNA NLRP3 (*P* ˃ 0.05, Fig. 4I). LncRNA NLRP3 expression was negatively correlated with miR-138-5p expression in LPS-treated NR8383 AM cells (Fig. 4J). Functionally, we found that lncRNA NLRP3 might compete for sponging miR-138-5p to regulate the NLRP3-triggered inflammatory response during LPS-induced ALI.

### miR-138-5p regulates the inflammatory response by targeting NLRP3

**Fig. 5:**
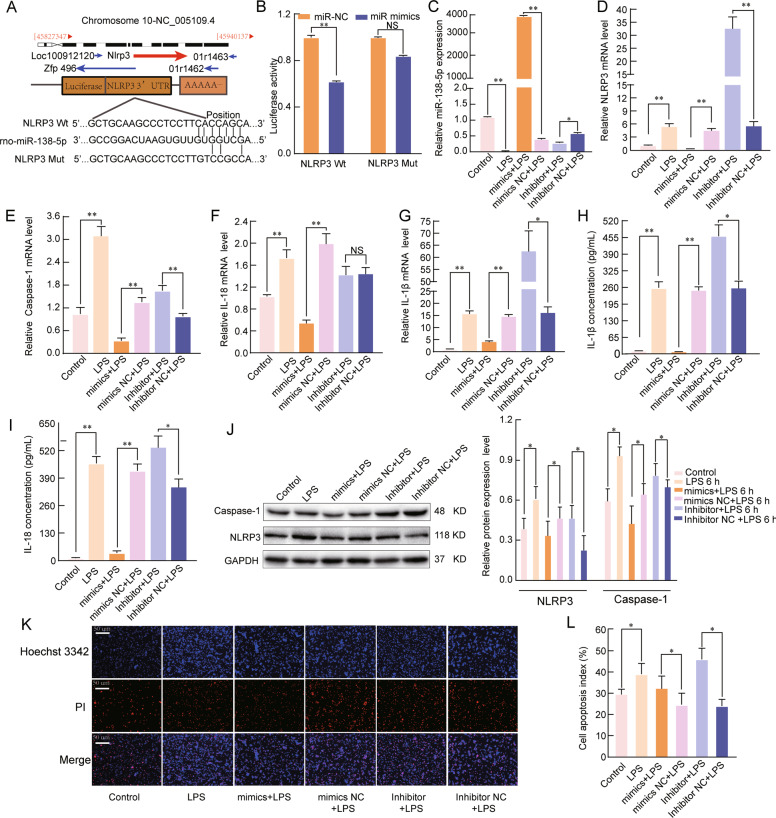
miR-138-5p regulates the inflammatory response by targeting NLRP3. **A** The predicted miR-138-5p-binding sites in the NLRP3 mRNA 3′-UTR. **B** A firefly luciferase reporter containing either wild-type or mutant NLRP3 was cotransfected into NR8383 AM cells with miR-138-5p mimics NC or miR-138-5p mimics. qRT-PCR assays were used to analyse the mRNA expression of **C** miR-138-5p, **D** NLRP3, **E** Caspase-1, **F** IL-18, and **G** IL-1β in the NR8383 AM cells (*n* = 6). β-Actin was used as a reference gene. **H**, **I** ELISA was used to analyse the IL-1β and IL-18 levels in the culture supernatants. **J** Western blotting assay of the protein expression levels of NLRP3 and Caspase-1. **K**, **L** Cell apoptosis was determined by Hoechst 33342 and PI dual staining assays (**K**) and counted (**L**). The data are presented as mean ± SE (*n* = 6). *P < 0.05; **P < 0.01; ***P < 0.001; NS, no statistically significant difference.

We next investigated whether miR-138-5p could regulate the inflammatory response associated with ALI by targeting NLRP3. Bioinformatic analysis showed that miR-138-5p directly binds to the 3′-UTR of NLRP3 (Fig. 5A). The luciferase reporter assay data indicated a noticeable decrease in the luciferase activity in the NLRP3-WT and miR-138-5p mimic cotransfected group compared to that in the NC group (*P* < 0.05, Fig. 5B). Next, we explored the expression of NLRP3, caspase-1, IL-18, and IL-1β in LPS-treated AM cells exposed to miR-138-5p mimics and inhibitors. The qRT-PCR results showed that the expression of NLRP3, caspase-1, IL-18, and IL-1β was significantly upregulated by miR-138-5p inhibition, and miR-138-5p mimics inhibited the mRNA expression of these genes (*P* < 0.05, Fig. 5C–G). The levels of IL-1β and IL-18 in the supernatants were measured by ELISA supported by the qRT-PCR findings (*P* < 0.05, Fig. 5H, I). In addition, miR-138-5p mimics reduced the NLRP3 and caspase-1 protein levels in LPS-treated AMs; however, inhibition of miR-138-5p expression increased the protein levels of NLRP3 and caspase-1 (*P* < 0.05, Fig. 5J). Hoechst 3342 staining showed that LPS-induced cell apoptosis was enhanced by the miR-138-5p inhibitor and reduced by the miR-138-5p mimic (*P* < 0.05, Fig. 5K, L). Taken together, these data indicated that miR-138-5p is a potential target gene of NLRP3 in LPS-induced ALI.

### LncRNA NLRP3 regulates the inflammatory response through the miR-138-5p/NLRP3/ IL-1β axis

**Fig. 6:**
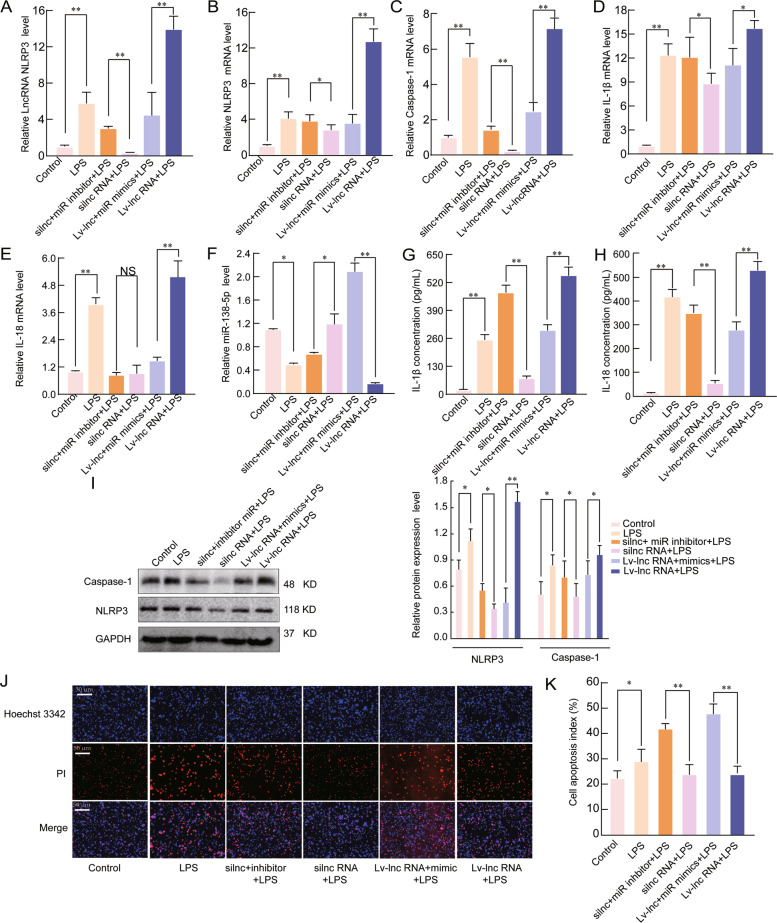
LncRNA NLRP3 regulates the inflammatory response through the lncRNA NLRP3/miR-138-5p/NLRP3 ceRNET in vitro. miR-138-5p suppression reversed the effects of silncRNA NLRP3 on the mRNA expression of **A** lncRNA NLRP3, **B** NLRP3, **C** Caspase-1, **D** IL-1β, **E** IL-18, and **F** miR-138-5p in NR8383 alveolar macrophage (AMs) cells. β-Actin was used as the reference gene. **G**, **H** ELISA analysis of the IL-1β and IL-18 levels in the culture supernatant. **I** Western blotting assay of the protein expression levels of NLRP3 and caspase-1. **J**, **K** Cell apoptosis was determined by Hoechst 33342 and PI dual staining assays (**J**) and counted (**K**). The data are presented as mean ± SE (*n* = 6). *P < 0.05; **P < 0.01; ***P < 0.001; NS, no statistically significant difference.

To further verify the role of the lncRNA NLRP3/miR-138-5p/NLRP3 axis, rescue assays were performed. qRT-PCR and western blotting assays showed that lncRNA NLRP3 silencing significantly decreased the expression of NLRP3 and its related inflammatory factors (caspase-1, IL-18, and IL-1β), whereas miR-138-5p suppression abolished these effects (*P* < 0.05, Fig. 6A–F, I). In addition, we showed that miR-138-5p mimics could reverse the effects of Lv-lncRNA NLRP3 on the NLRP3-triggered inflammatory response in LPS-induced ALI (*P* < 0.05, Fig. 6A–F, I). As expected, the miR-138-5p inhibitor markedly reversed the lncRNA NLRP3-mediated decrease in the levels of the inflammatory factors IL-18 and IL-1β in the supernatant. In contrast, miR-138-5p mimics reversed the effects of lncRNA NLRP3 overexpression (*P* < 0.05, Fig. 6G, H). Furthermore, miR-138-5p mimics weakened the effects of Lv-lncRNA NLRP3 on the protein levels of caspase-1 in LPS-treated AM cells; however, inhibition of miR-138-5p reversed the effects of lncRNA NLRP3 silencing on the protein levels of NLRP3 and caspase-1 (*P* < 0.05, Fig. 6I). Similarly, Hoechst 3342 staining showed that LPS-induced cell apoptosis was enhanced when the cells were transfected with Lv-lncRNA NLRP3 + miR-138-5p mimics compared to Lv-lncRNA NLRP3 and when the cells were transfected with silncRNA NLRP3 + miR-138-5p inhibitor compared to silncRNA NLRP3 (*P* < 0.05, Fig. 6J, K). Thus, we suggest that lncRNA NLRP3 promotes NLRP3 inflammasome activation through the miR-138-5p/NLRP3/IL-1β axis in LPS-induced ALI.

### LncRNA NLRP3 functions in the ceRNET during the NLRP3-triggered inflammatory response in vivo

**Fig. 7:**
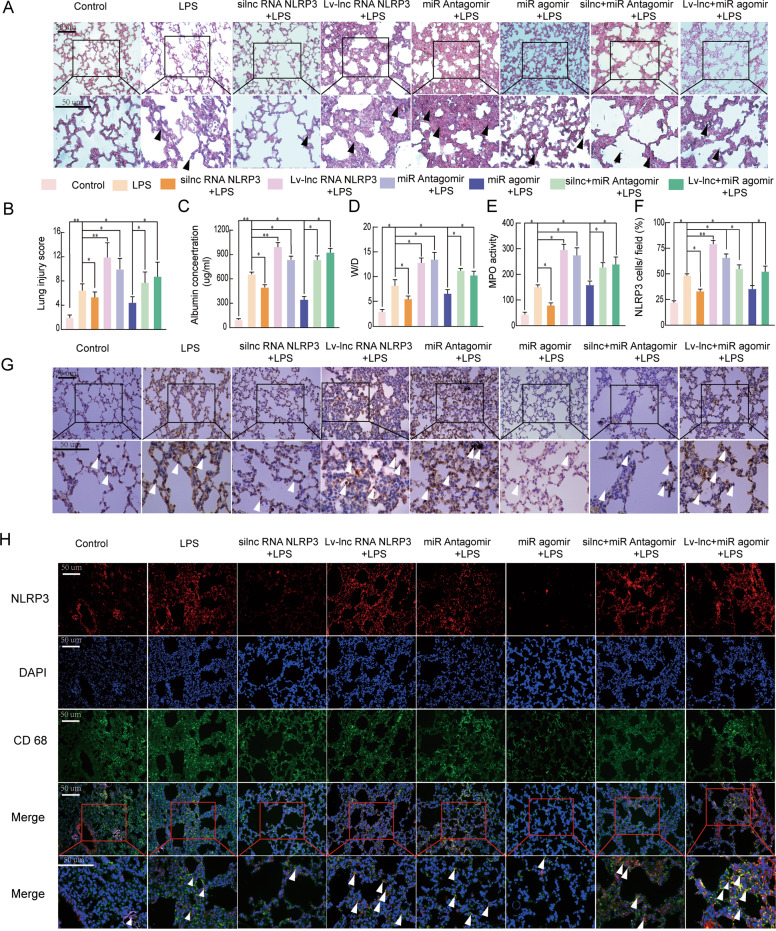
LncRNA NLRP3/miR-138-5p/NLRP3 functions via the ceRNET during the NLRP3-triggered inflammatory response in vivo. Rat lungs were injected with PBS in the control group and LPS-treated rats were further treated with si-r-lncRNA NLRP3, Lv-lncRNA NLRP3, agomiR-138-5p, antagomiR-138-5p, Lv-lncRNA NLRP3 + agomiR-138-5p, and si-r-lncRNA NLRP3 + antagomiR-138-5p. **A** Lung tissue samples were collected 6 h after establishing LPS-induced ALI to analyse the histopathological changes (×200, ×400). The black arrow indicates neutrophil infiltration, pulmonary oedema, alveolar wall thickening, and alveolar haemorrhage. **B** The lung injury score was determined via H&E staining, a representative histological analysis (*n* = 6 animals per group). **C** ELISA was used to measure the BALF albumin content. **D** Detection of the lung W/D ratio in rats. **E** MPO activity in the lung tissues of rats. **F**, **G** Immunohistochemical detection of the NLRP3 contents in rat lung tissues (×200, ×400). **H** The inflammatory response in NR8383 AM cells was suppressed by si-r-lncRNA NLRP3 and miR-138-5p mimics alone or in combination, as shown by the decreased number of cells colabeled with CD68 (green) and NLRP3 (red). LncRNA NLRP3 overexpression, miR-138-5p inhibition, and NLRP3 augmented the inflammatory response in LPS-induced ALI with more NLRP3 and CD68 anchored in the plasma membrane of the AM cells. The data are presented as mean ± SE (*n* = 6). *P < 0.05; **P < 0.01; ***P < 0.001; NS, no statistically significant difference.

To investigate the lncRNA NLRP3/miR-138-5p/NLRP3 axis in vivo and explore the underlying regulatory mechanism, LPS-induced ALI animal models were established. Typical pathological changes in the lungs, such as neutrophil infiltration, pulmonary oedema, alveolar wall thickening, and alveolar haemorrhage, were observed in each group after the LPS challenge. The degree of each characteristic was scored as 3 (prominent), 2 (moderate), 1 (mild), and 0 (absence). However, si-r-lncRNA NLRP3 administration ameliorated the histopathological changes associated with LPS-induced ALI (*P* < 0.05, Fig. 7A, B). Compared with those in the NC group (PBS), the BALF albumin levels, W/D, and MPO were decreased in the si-r-lncRNA NLRP3-treated group (*P* < 0.05, Fig. 7C–E). Moreover, the expression of NLRP3 was analysed in the lung tissues from rats by IHC and IF staining. The inflammatory response in ALI was significantly decreased in both the si-r-lncRNA NLRP3 and agomiR-138-5p groups compared to the control group (PBS). Silencing of lncRNA NLRP3 and treatment with antagomiR-138-5p led to reduced inflammatory responses, as shown by IHC staining in the LPS-induced ALI model. The protective effect was eliminated by Lv-lncRNA NLRP3 overexpression and antagomiR-138-5p markedly increased the number of activated AM cells in ALI (*P* < 0.05, Fig. 7F, G). The inflammatory response in LPS-induced ALI was significantly suppressed by si-r-lncRNA NLRP3 and agomiR-138-5p alone or in combination, as shown by the reduced number of CD68 and NLRP3 double-positive cells. LncRNA NLRP3 overexpression and miR-138-5p knockdown enhanced the LPS-induced inflammatory response in ALI (*P* < 0.05, Fig. 7H). The results described above showed that lncRNA NLRP3 promoted NLRP3 inflammasome activation through the miR-138-5p/NLRP3/IL-1β axis in the LPS-induced ALI and these phenomena are also observed in vivo.

### The mechanism by which the lncRNA NLRP3/miR-138-5p/NLRP3 ceRNET functions in ALI

**Fig. 8:**
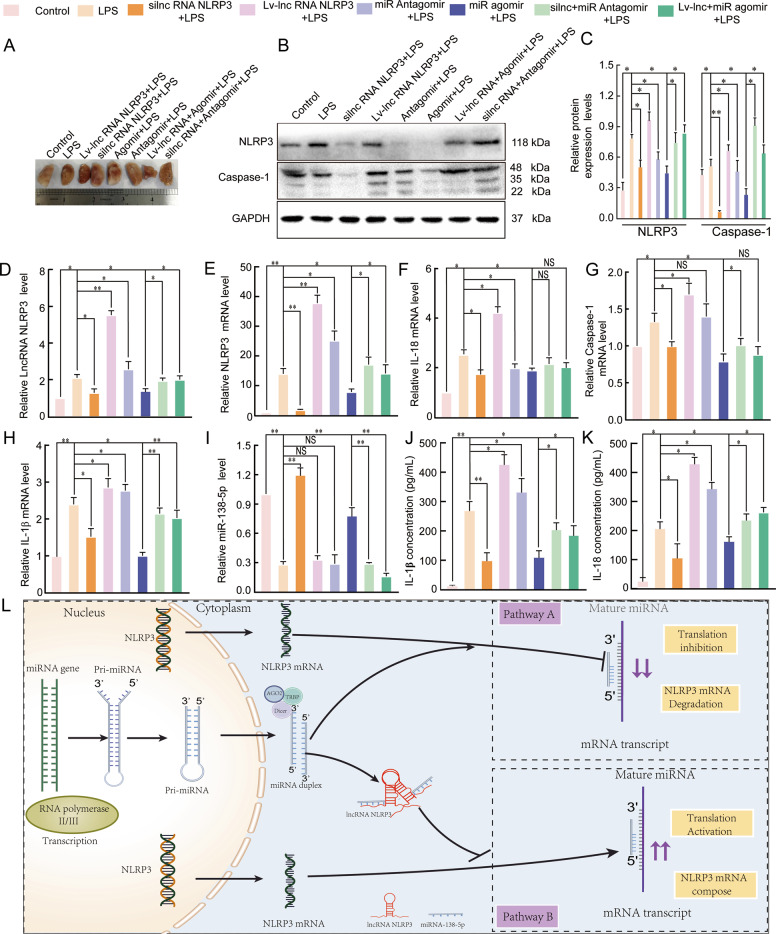
The mechanism by which the lncRNA NLRP3/miR-138-5p/NLRP3 ceRNET functions in the inflammatory response. The lungs of rats were injected with PBS in the control group and LPS-treated rats were further treated with si-r-lncRNA NLRP3, Lv-lncRNA NLRP3, agomiR-138-5p, antagomiR-138-5p, Lv-lncRNA NLRP3 + agomiR-138-5p, and si-r-lnc NLRP3 + antagomiR-138-5p. **A** Morphometric changes in the appearance of the lungs that had been fixed in 4% paraformaldehyde for 24 h at 25 °C in each group. **B**, **C** The protein expression levels of NLRP3 and caspase-1 in rat lung tissues. qRT-PCR assays were used to analyse mRNA expression of **D** lncRNA NLRP3, **E** NLRP3, **F** IL-18, **G** Caspase-1, **H** IL-1β, and **I** miR-138-5p in the lung tissues of rats. ELISA analysis of the IL-1β (**J**) and IL-18 (**K**) levels in the culture supernatant. **L** Graphical summary of the role of the lncRNA NLRP3/miR-138-5p/NLRP3 ceRNET in acute lung injury. β-Actin was used as the reference. The data are presented as mean ± SE (*n* = 6). *P < 0.05; **P < 0.01; ***P < 0.001; NS, no statistically significant difference.

Finally, we investigated the mechanism by which the lncRNA NLRP3/miR-138-5p/NLRP3 ceRNET functions in the inflammatory response of ALI. We observed morphological changes in the lung tissue samples of the rats in each group after they were fixed in 4% paraformaldehyde for 24 h (25 °C). The lesions in the lung tissues from the Lv-lncRNA and antagomir groups were the most severe, those from the agomiR and si-r-lncRNA groups were mild, and those from the agomiR + Lv-lncRNA, antagomir + si-r-lncRNA, and LPS groups were moderate (Fig. 8A). The expression of lncRNA NLRP3, NLRP3, miR-138-5p, caspase-1, IL-18, and IL-1β in LPS-induced ALI was examined by qRT-PCR and western blotting was used to examine NLRP3 and caspase-1 protein expression. NLRP3 and caspase-1 protein expression and lncRNA NLRP3, NLRP3, caspase-1, IL-18, and IL-1β mRNA expression were decreased in the si-r-lncRNA NLRP3 group, and antagomir-138-5p reversed these effects. However, the protein and mRNA expression of these molecules was increased in the Lv-lncRNA NLRP3 group and agomir-138-5p abolished these effects. Under the same conditions, miR-138-5p expression exhibited the opposite trend to the mRNA expression trends described above (*P* < 0.05, Fig. 8B–I). In addition, the levels of IL-18 and IL-1β in the supernatants of tissues from rats with LPS-induced ALI were examined by ELISA. The levels of IL-18 and IL-1β supported the results of the mRNA measurements (*P* < 0.05, Fig. 8J, K). Therefore, a graphical summary of the role of the lncRNA NLRP3/miR-138-5p/NLRP3 ceRNET in ALI is presented in Fig. 8L. In LPS-treated AM cells, increased lncRNA NLRP3 expression, decreased miR-138-5p expression, increased NLRP3 inflammasome activation, and enhanced inflammatory responses were observed during ALI. LncRNA NLRP3 acted as a sponge for miR-138-5p to upregulate NLRP3 expression, leading to NLRP3 inflammasome activation and subsequent IL-18 and IL-1β induction in AM cells during ALI. These results indicated that the lncRNA NLRP3/miR-138-5p/NLRP3 ceRNET exerted pivotal regulatory effects on the inflammatory response in ALI.

## Discussion

ALI and ARDS are continuous lung changes that arise from the inflammatory response or other types of lung injuries, and these conditions frequently result in significant morbidity or death [43]. The absence of known triggers of injury and validated therapeutic targets has restricted the effective treatment of ALI and ARDS. Therefore, exploring new molecules and molecular regulatory mechanisms in ALI has become an important direction to improve its treatment. Our study includes the following four main findings. First, we confirmed that lncRNA NLRP3, miR-138-5p, and NLRP3 have crucial roles in the onset of ALI through RNA-seq and bioinformatics analyses. Second, we validated the identification and functional characterization of the lncRNA NLRP3/miR-138-5p/NLRP3 ceRNET, which provides a novel direction for thinking about the inflammatory response in ALI. Third, NLRP3 inflammasome activation expands our deep understanding and treating immune-related inflammatory responses. Fourth, the NLRP3-triggered inflammatory response was confirmed to be pivotal in early ALI.

The newly discovered lncRNA NLRP3 is one of the most highly expressed lncRNAs in response to LPS treatment and the molecular mechanisms underlying the relationship between NLRP3 and lncRNA NLRP3 were determined in this study through bioinformatics analysis. Furthermore, the miR-138-5p expression was decreased after LPS treatment [31]. We have demonstrated the functions of and miR-138-5p and lncRNA NLRP3 in regulating AM inflammation during ALI. Knockdown of lncRNA NLRP3 was accompanied by a substantial increase in the LPS-stimulated expression of miR-138-5p and decreased expression of NLRP3, caspase-1, IL-18, and IL-1β in vivo and in vitro. These results show that lncRNA LRP3, NLRP3, and miR-138-5p may initiate a new mechanism through ceRNET, thus regulating NLRP3 inflammasome activation. Mechanistically, lncRNA NLRP3 increased in LPS-induced ALI and sponged miR-138-5p to facilitate NLRP3 expression. LncRNAs sponging multiple miRNAs to regulate the expression of NLRP3 inflammasome components have been found to play a role in the progression and occurrence of various diseases [14, 17, 19, 21, 23, 44–46]. However, the function of non-coding lncRNA NLRP3 and miR-138-5p in ALI has still not been reported. According to the bioinformatics analysis, it was predicted that miR-138-5p might be a target of lncRNA NLRP3 and NLRP3. miR-138-5p has been found to be a metastatic and tumour suppressor in different types of diseases [25–29]. Our previous study found that miR-138-5p plays a vital role in neuroinflammation by targeting NLRP3 and this phenomenon can cause cognitive impairment [30]. The NLRP3 protein is a crucial element of the multifunctional inflammasome complex in the inflammatory signalling pathway. The NLRP3 inflammasome that participates in the innate immune response is a major intracellular inflammatory pathway [47]. Therefore, as essential hub genes in early ALI, lncRNA NLRP3 and miR-138-5p are likely to be promising therapeutic targets with significant efficacy, because they can both regulate NLRP3 activation. Moreover, lncRNA NLRP3 can upregulate NLRP3 expression through the regional delivery of siRNA or gene editing. Thus, we can use multiple mechanisms involved to inhibit NLRP3 activation. This evidence also provides insights for developing therapeutics, i.e., exploring the hub genes that are central in pathogenesis, facilitating prophylaxis, and treating other complex diseases.

Our results suggested that the ceRNET, in addition to traditional protein-coding-centric studies, is a non-coding regulatory mechanism that has two significant advantages. First, the ceRNET responds to lung damage at an earlier stage of ALI. Transcriptional regulation mechanisms are more effective than translational regulation mechanisms on the RNA expression level. Second, the ceRNET regulation mechanism represents a robust scaffolding for transcription based on competitive miRNAs. Cumulative evidence has shown that lncRNAs are implicated in various pathophysiological processes, such as ageing, tumours, cardiovascular disease, and neurodegenerative disease [14, 17, 19–23, 45, 46, 48–53]. To date, there are six types of regulatory mechanisms between lncRNA and NLRP3 [24]. DNA methyltransferases of lncRNAs affect NLRP3 chromatin reconstruction and modifications [16]. LncRNAs have an essential role in NLRP3 inflammasome activation via the NF-κB/NLRP3 inflammasome pathway [54]. Broker et al. [53] found that Gm15441 expression suppressed its antisense transcription and encoded thioredoxin interacting protein (TXNIP), which downregulated TXNIP-triggered NLRP3 inflammasome activity. Moreover, researchers have found that Neat1 enhances the assembly of many canonical inflammasomes and promotes caspase-1 protease activity and caspase-1 secretion [49]. We confirmed that lncRNA NLRP3 sponged miR-138-5p to upregulate NLRP3 expression, strengthening the ability of macrophages to increase inflammatory cytokine expression in ALI. Novel molecular lncRNAs that modulate NLRP3 expression are involved in regulating macrophage immune responses in ALI. However, further investigation is required to elucidate new mechanisms of interaction between lncRNA NLRP3 and the NLRP3 inflammasome in addition to the ceRNET mechanism in ALI.

Notably, our finding regarding NLRP3 as a trigger of the early inflammatory response can enhance our understanding of ALI. The most fully characterized inflammasome-associated proteins (including caspase-1 and NLRP3) were measured in the present study to evaluate inflammasome activity. Our results show that the increased expressions of caspase-1, IL-1β, IL-18, and the NLRP3 inflammasome were positively associated with lung structural damage, with the functional deteriorations in ALI. Numerous studies have revealed that activation of NLRP3 inflammasome in macrophages cells can sniff cellular damage [49, 55] and modulates inflammatory responses in cognitive impairment [44, 56], ALI [42], cancer [50], renal disease [21, 22], liver disease [55], and diabetes mellitus [45, 46]. Cumulative evidence has confirmed that the NLRP3 inflammasome was involved in the inflammatory responses of ALI established by LPS-induced AM and indicated that its suppression could alleviate ALI [7]. Activated NLRP3 initiates maturation and secretion of cytokines (IL-1β and IL-18) and pyroptosis must also be considered together as a potential inflammatory mechanism, which triggers a form of AM pyroptosis [57]. These results can provide profound insight for understanding NLRP3-triggered inflammatory storms and treating NLRP3-related immunological diseases.

Collectively, our study reveals that lncRNA NLRP3 modulates the expression of the proinflammatory cytokines in LPS-induced ALI through an NLRP3-mediated ceRNET-dependent mechanism. LncRNA NLRP3 promotes activation of the NLRP3 inflammasome by binding to miR-138-5p resulting in IL-18 and IL-1β secretion. These results provide new molecular mechanisms by which lncRNAs and miRNAs regulate NLRP3 inflammasome activation in ALI. Our findings indicate that the interaction between lncRNAs and NLRP3 provides insights into the treatment of early ALI.

## Supplementary information

Supplementary Table 1

Supplementary data

## Data Availability

The data that support the findings of this study are available on request from the corresponding author.

## References

[1] Sercundes MK, Ortolan LD, Debone D, Aitken EH, Alvarez JM, Russo M, et al. Inflammatory factors and leucocytes are involved in the pathogenesis of malaria associated acute lung injury/acute respiratory distress syndrome in murine model. Front Immunol. 2013;4.

[2] Ostwani W, Shanley TP (2014). Acute lung injury (ALI) and acute respiratory distress syndrome (ARDS). Pediatr Crit Care Med.

[3] Lord JM, Midwinter MJ, Chen Y-F, Belli A, Brohi K, Kovacs EJ (2014). The systemic immune response to trauma: an overview of pathophysiology and treatment. Lancet.

[4] Rosadini CV, Kagan JC (2017). Early innate immune responses to bacterial LPS. Curr Opin Immunol.

[5] Tartey S, Takeuchi O (2017). Pathogen recognition and Toll-like receptor targeted therapeutics in innate immune cells. Int Rev Immunol.

[6] Alarcón MML, Ruocco JF, Ferreira F, Paula-Neto HA, Sepúlveda M, Vila Petroff M (2018). Toll-like receptor 4 and NLRP3 caspase 1-interleukin-1β-axis are not involved in colon ascendens stent peritonitis-associated heart disease. Shock.

[7] Wu J, Yan Z, Schwartz DE, Yu J, Malik AB, Hu G (2013). Activation of NLRP3 inflammasome in alveolar macrophages contributes to mechanical stretch-induced lung inflammation and injury. J Immunol.

[8] De Nardo D, Latz E (2011). NLRP3 inflammasomes link inflammation and metabolic disease. Trends Immunol.

[9] Jo E-K, Kim JK, Shin D-M, Sasakawa C (2016). Molecular mechanisms regulating NLRP3 inflammasome activation. Cell Mol Immunol.

[10] Yang Y, Wang H, Kouadir M, Song H, Shi F (2019). Recent advances in the mechanisms of NLRP3 inflammasome activation and its inhibitors. Cell Death Dis.

[11] Grailer JJ, Canning BA, Kalbitz M, Haggadone MD, Dhond RM, Andjelkovic AV (2014). Critical role for the NLRP3 inflammasome during acute lung injury. J Immunol.

[12] Jiang L, Zhang L, Kang K, Fei D, Gong R, Cao Y (2016). Resveratrol ameliorates LPS-induced acute lung injury via NLRP3 inflammasome modulation. Biomed Pharmacother.

[13] Khan S, Masood M, Gaur H, Ahmad S, Syed MA (2021). Long non-coding RNA: An immune cells perspective. Life Sci.

[14] Yu SY, Dong B, Tang L, Zhou SH (2018). LncRNA MALAT1 sponges miR-133 to promote NLRP3 inflammasome expression in ischemia-reperfusion injured heart. Int J Cardiol.

[15] Wu L-M, Wu S-G, Chen F, Wu Q, Wu C-M, Kang C-M (2020). Atorvastatin inhibits pyroptosis through the lncRNA NEXN-AS1/NEXN pathway in human vascular endothelial cells. Atherosclerosis.

[16] She Q, Shi P, Xu S-S, Xuan H-Y, Tao H, Shi K-H (2020). DNMT1 methylation of LncRNA GAS5 leads to cardiac fibroblast pyroptosis via affecting NLRP3 axis. Inflammation.

[17] Zhang X, Wu N, Wang J, Li Z (2019). LncRNA MEG3 inhibits cell proliferation and induces apoptosis in laryngeal cancer via miR‐23a/APAF‐1 axis. Biomed Pharmacother.

[18] Zhang M, Zheng Y, Sun Y, Li S, Chen L, Jin X (2019). Knockdown of NEAT1 induces tolerogenic phenotype in dendritic cells by inhibiting activation of NLRP3 inflammasome. Theranostics.

[19] Yu L, Hao Y, Xu C, Zhu G, Cai Y (2019). LINC00969 promotes the degeneration of intervertebral disk by sponging miR‐335‐3p and regulating NLRP3 inflammasome activation. IUBMB Life.

[20] Xue Z, Zhang Z, Liu H, Li W, Guo X, Zhang Z (2019). lincRNA-Cox2 regulates NLRP3 inflammasome and autophagy mediated neuroinflammation. Cell Death Differ.

[21] Song Z, Zhang Y, Gong B, Xu H, Hao Z, Liang C (2019). Long noncoding RNA LINC00339 promotes renal tubular epithelial pyroptosis by regulating the miR‐22‐3p/NLRP3 axis in calcium oxalate–induced kidney stone. J Cell Biochem.

[22] Hu J, Wu H, Wang D, Yang Z, Dong J (2019). LncRNA ANRIL promotes NLRP3 inflammasome activation in uric acid nephropathy through miR-122-5p/BRCC3 axis. Biochimie.

[23] Hu H, Wang Y, Ding X, He Y, Lu Z, Wu P (2018). Long non-coding RNA XLOC_000647 suppresses progression of pancreatic cancer and decreases epithelial-mesenchymal transition-induced cell invasion by down-regulating NLRP3. Mol Cancer.

[24] Luo D, Liu F, Zhang J, Shao Q, Tao W, Xiao R (2021). Functional crosstalk between Long non-coding RNAs and the NLRP3 inflammasome in the regulation of diseases. Mol Immunol.

[25] Rong Y, Liu M, Liang H, Guo S, Zhang C (2016). miR-138-5p contributes to cell proliferation and invasion by targeting Survivin in bladder cancer cells. Mol Cancer.

[26] Gao Y, Fan XW, Li WN, Ping W, Deng Y, Fu XN (2014). miR-138-5p reverses gefitinib resistance in non-small cell lung cancer cells via negatively regulating G protein-coupled receptor 124. Biochem Biophys Res Commun.

[27] Lian Z, Yu H, Yi S, Peng X, Shen S (2016). The tumor suppressor miR-138-5p targets PD-L1 in colorectal cancer. Oncotarget.

[28] Wu H, Wang C, Liu Y, Yang C, Li X (2020). miR-138-5p suppresses glioblastoma cell viability and leads to cell cycle arrest by targeting cyclin D3. Oncol Lett.

[29] Yu C, Wang M, Li Z, Xiao J, Peng F, Guo X (2015). MicroRNA-138-5p regulates pancreatic cancer cell growth through targeting FOXC1. Cell Oncol.

[30] Feng X, Hu J, Zhan F, Luo D, Hua F, Xu G (2021). MicroRNA-138-5p regulates hippocampal neuroinflammation and cognitive impairment by NLRP3/caspase-1 signaling pathway in rats. J Inflamm Res.

[31] Luo D, Liu F, Zhang J, Shao Q, Qian K (2021). Comprehensive analysis of LncRNA-mRNA expression profiles and the ceRNA network associated with pyroptosis in LPS-induced acute lung injury. J Inflamm Res.

[32] Ahmad A, Lin H, Shatabda S (2020). Locate-R: subcellular localization of long non-coding RNAs using nucleotide compositions. Genomics.

[33] Chen YT, Du Y, Zhao B, Gan LX, Yu KK, Sun L (2019). Costunolide alleviates HKSA-induced acute lung injury via inhibition of macrophage activation. Acta Pharm Sin.

[34] Chang TH, Huang HY, Hsu JB, Weng SL, Horng JT, Huang HD (2013). An enhanced computational platform for investigating the roles of regulatory RNA and for identifying functional RNA motifs. BMC Bioinformatics.

[35] Healy J, Dionne J, Bélanger H, Lariviere M, Beaulieu P, Labuda D (2009). Functional impact of sequence variation in the promoter region of TGFB1. Int J Cancer.

[36] Bushehri A, Barez MM, Mansouri S, Biglarian A, Ohadi M (2016). Genome-wide identification of human-and primate-specific core promoter short tandem repeats. Gene.

[37] Agarwal V, Bell GW, Nam J-W, Bartel DP (2015). Predicting effective microRNA target sites in mammalian mRNAs. eLife.

[38] Kozomara A, Birgaoanu M, Griffiths-Jones S (2019). miRBase: from microRNA sequences to function. Nucleic Acids Res.

[39] Li J-H, Liu S, Zhou H, Qu L-H, Yang J-H (2014). starBase v2. 0: decoding miRNA-ceRNA, miRNA-ncRNA and protein–RNA interaction networks from large-scale CLIP-Seq data. Nucleic Acids Res.

[40] Mann M, Wright PR, Backofen R (2017). IntaRNA 2.0: enhanced and customizable prediction of RNA–RNA interactions. Nucleic Acids Res.

[41] Kellum JA, Kramer DJ, Lee K, Mankad S, Bellomo R, Pinsky MR (1997). Release of lactate by the lung in acute lung injury. Chest..

[42] Lin Y, Yang Y (2019). MiR-24 inhibits inflammatory responses in LPS-induced acute lung injury of neonatal rats through targeting NLRP3. Pathol Res Pr.

[43] Butt Y, Kurdowska A, Allen TC (2016). Acute lung injury: a clinical and molecular review. Arch Pathol Lab Med.

[44] Haque ME, Akther M, Jakaria M, Kim IS, Azam S, Choi DK (2020). Targeting the microglial NLRP3 inflammasome and its role in Parkinson’s disease. Mov Disord.

[45] Che H, Wang Y, Li H, Li Y, Sahil A, Lv J (2020). Melatonin alleviates cardiac fibrosis via inhibiting lncRNA MALAT1/miR-141-mediated NLRP3 inflammasome and TGF-beta1/Smads signaling in diabetic cardiomyopathy. FASEB J.

[46] Song Y, Yang L, Guo R, Lu N, Shi Y, Wang X (2019). Long noncoding RNA MALAT1 promotes high glucose-induced human endothelial cells pyroptosis by affecting NLRP3 expression through competitively binding miR-22. Biochem Bioph Res Commun.

[47] Zhong Y, Lu Y, Yang X, Tang Y, Zhong X (2020). The roles of NLRP3 inflammasome in bacterial infection. Mol Immunol.

[48] Zhang X, Li DY, Reilly MP (2019). Long intergenic noncoding RNAs in cardiovascular diseases: challenges and strategies for physiological studies and translation. Atherosclerosis.

[49] Zhang P, Cao L, Zhou R, Yang X, Wu M (2019). The lncRNA Neat1 promotes activation of inflammasomes in macrophages. Nat Commun.

[50] Xu Z, Xi K (2019). LncRNA RGMB-AS1 promotes laryngeal squamous cell carcinoma cells progression via sponging miR-22/NLRP3 axis. Biomed Pharmacother.

[51] Ma M, Pei Y, Wang X, Feng J, Zhang Y, Gao MQ (2019). LncRNA XIST mediates bovine mammary epithelial cell inflammatory response via NF‐κB/NLRP3 inflammasome pathway. Cell Prolif.

[52] de Oliveira JC, Oliveira LC, Mathias C, Pedroso GA, Lemos DS, Salviano‐Silva A (2019). Long non‐coding RNAs in cancer: another layer of complexity. J Gene Med.

[53] Brocker C, Kim D, Melia T, Karri K, Gonzalez FJ (2020). Long non-coding RNA Gm15441 attenuates hepatic inflammasome activation in response to PPARA agonism and fasting. Nat Commun.

[54] Yi H, Peng R, Zhang L-Y, Sun Y, Peng H-M, Liu H-D (2017). LincRNA-Gm4419 knockdown ameliorates NF-κ B/NLRP3 inflammasome-mediated inflammation in diabetic nephropathy. Cell Death Dis.

[55] Zhang K, Shi Z, Zhang M, Dong X, Zheng L, Li G (2020). Silencing lncRNA Lfar1 alleviates the classical activation and pyoptosis of macrophage in hepatic fibrosis. Cell Death Dis.

[56] Wan P, Su W, Zhang Y, Li Z, Deng C, Li J (2019). LncRNA H19 initiates microglial pyroptosis and neuronal death in retinal ischemia/reperfusion injury. Cell Death Differ.

[57] Liu X, Zhang X, Ding Y, Zhou W, Tao L, Lu P (2016). Nuclear factor E2-related factor-2 negatively regulates NLRP3 inflammasome activity by inhibiting reactive oxygen species-induced NLRP3 priming. Antioxid Redox Signal.

