# Supplementary material for: Suppression of lncRNA NLRP3 inhibits NLRP3-triggered inflammatory responses in early acute lung injury
Source: Cell Death Dis. 2021 Oct 1;12(10):898. doi: 10.1038/s41419-021-04180-y (PMC8486756; doi:10.1038/s41419-021-04180-y)
Supplement: Supplementary file 1 — Supplementary Table 1 [file 41419_2021_4180_MOESM1_ESM.docx]

Supplementary Table 1. The primers of lncRNA and mRNAs for qRT-PCR.

| LncRNA and mRNAs | Primer (5’-3’) |
| --- | --- |
| β-actin | Forward: TACTGCCCTGGCTCCTAGCA  Reverse: TGGACAGTGAGGCCAGGATAG |
| Caspase-1 | Forward: AACACCCACTCGTACACGTC  Reverse: TGAGGTCAACATCAGCTCCG |
| miR-138-5p | Forward: GGCCGGACTAAGTGTTGT  Reverse: GCAGGGTCCGAGGTATTC |
| IL-1β | Forward: ATGAGGACCCAAGCACCTTC  Reverse: ACCACTTGTTGGCTTATGTTCTG |
| IL-18 | Forward: AAAGTGCCAGTGAACCC  Reverse: TTTGATGTAAGTTAGTGAGAGTGA |
| NLRP3 | Forward: AACTTGCAGAAGCTGGGGTT  Reverse: GGTGCAGAAGTCCCTCACAG |
| LncRNA NLRP3 | Forward: CTTGGAGGATAGAGGCTCTTTTCTT  Reverse: AGGTCGTTGTTGCTCAAGTTCAG |
